# *PIK3CA* mutations in non-small cell lung cancer (NSCLC): Genetic heterogeneity, prognostic impact and incidence of prior malignancies

**DOI:** 10.18632/oncotarget.2834

**Published:** 2014-11-26

**Authors:** Matthias Scheffler, Marc Bos, Masyar Gardizi, Katharina König, Sebastian Michels, Jana Fassunke, Carina Heydt, Helen Künstlinger, Michaela Ihle, Frank Ueckeroth, Kerstin Albus, Monika Serke, Ulrich Gerigk, Wolfgang Schulte, Karin Töpelt, Lucia Nogova, Thomas Zander, Walburga Engel-Riedel, Erich Stoelben, Yon-Dschun Ko, Winfried Randerath, Britta Kaminsky, Jens Panse, Carolin Becker, Martin Hellmich, Sabine Merkelbach-Bruse, Lukas C. Heukamp, Reinhard Büttner, Jürgen Wolf

**Affiliations:** ^1^ Center for Integrated Oncology Köln Bonn, Cologne, Germany; ^2^ Lung Cancer Group Cologne, Department I for Internal Medicine, University Hospital of Cologne, Cologne, Germany; ^3^ Institute of Pathology, University Hospital of Cologne, Cologne, Germany; ^4^ Department for Pulmonology and Thoracic Oncology, Lung Clinic Hemer, Hemer, Germany; ^5^ Clinic for Hematology, Oncology and Palliative Care, Malteser Hospital, Bonn, Germany; ^6^ Gastrointestinal Cancer Group Cologne, Department I for Internal Medicine, University Hospital of Cologne, Cologne, Germany; ^7^ Lung Clinic Merheim, Hospital of Cologne, Cologne, Germany; ^8^ Johanniter Hospital, Evangelical Clinics of Bonn, Bonn, Germany; ^9^ Clinic for Pneumology and Allergology Center for Sleep Medicine and Respiratory Care, Bethanien Hospital, Solingen, Germany; ^10^ Department of Medicine IV, University Hospital RWTH Aachen, Aachen, Germany; ^11^ Institute of Medical Statistics, Informatics, and Epidemiology, University of Cologne, Cologne, Germany

**Keywords:** Non-small cell lung cancer, PIK3CA, mutation, lung cancer, PI3K

## Abstract

Background: Somatic mutations of the *PIK3CA* gene have been described in non-small cell lung cancer (NSCLC), but limited data is available on their biological relevance. This study was performed to characterize *PIK3CA*-mutated NSCLC clinically and genetically.

Patients and methods: Tumor tissue collected consecutively from 1144 NSCLC patients within a molecular screening network between March 2010 and March 2012 was analyzed for *PIK3CA* mutations using dideoxy-sequencing and next-generation sequencing (NGS). Clinical, pathological, and genetic characteristics of *PIK3CA*-mutated patients are described and compared with a control group of *PIK3CA*-wildtype patients.

Results: Among the total cohort of 1144 patients we identified 42 (3.7%) patients with *PIK3CA* mutations in exon 9 and exon 20. These mutations were found with a higher frequency in sqamous cell carcinoma (8.9%) compared to adenocarcinoma (2.9%, p<0.001). The most common *PIK3CA* mutation was exon 9 E545K. The majority of patients (57.1%) had additional oncogenic driver aberrations. With the exception of *EGFR*-mutated patients, non of the genetically defined subgroups in this cohort had a significantly better median overall survival. Further, *PIK3CA*-mutated patients had a significantly higher incidence of malignancy prior to lung cancer (p<0.001).

Conclusion: *PIK3CA*-mutated NSCLC represents a clinically and genetically heterogeneous subgroup in adenocarcinomas as well as in squamous cell carcinomas with a higher prevalence of these mutations in sqamous cell carcinoma. *PIK3CA* mutations have no negative impact on survival after surgery or systemic therapy. However, PIK3CA mutated lung cancer frequently develops in patients with prior malignancies.

## INTRODUCTION

Non-small cell lung cancer (NSCLC) remains the most common cause of cancer-related death in the western world [1]. Nevertheless, the identification of therapeutically targetable driver mutations like activating mutations in the epidermal growth factor receptor (*EGFR*) and rearrangements of the *ALK* oncogene and the subsequent introduction of personalized treatment approaches has improved outcome of these genetically defined NSCLC subgroups within the last decade [1-5]. Thus, to date the identification and clinical evaluation of further targetable oncogenes is a major goal in lung cancer research [6, 7].

**Figure 1:**
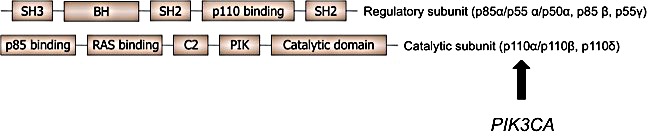
Schematic figure of the PI3-Kinase The catalytic subunit p110α is encoded by the *PIK3CA* gene on chromosome 3q26.3. Activating mutations in PI3 kinase are considered oncogenic and targetable.

The phosphatidylinositol 3-kinases (PI3K) play a pivotal role in cell metabolism and proliferation, affecting both cancer and metabolic disorders [8-10]. Mutations in the *PIK3CA* gene encoding the class I PI3K p110α, are commonly found in a variety of cancers (Figure 1) [11]. In NSCLC, mutations within *PIK3CA* usually affecting the helical binding domain (exon 9, E545K or E542K) or the catalytic subunit (exon 20, H1047R or H1047L), are considered oncogenic and targetable [7, 12-16]. However, in some contrast to classical oncogenic driver mutations like activating *EGFR* mutations, *PIK3CA* mutations in adenocarcinomas of the lung (AD) have not been described to be mutually exclusive. Rather, co-occurrence with aberrations in *EGFR*, *BRAF*, *ALK* and, most frequently, *KRAS* were found. This observation raises the question whether the *PIK3CA* mutation alone is a sufficient oncongenic driver in NSCLC tumor formation [17-21]. Accordingly, specific PI3K inhibition in solid tumors did not lead to impressive response rates in mutated patients yet [22]. Only limited information is available so far on *PIK3CA* mutations in squamous cell lung cancer (SQCC) [23]. Recently, in a small series 4 patients with SQCC and *PIK3CA* mutations have been identified resulting in a frequency of 4.2% in the described cohort [24].

NSCLC subgroups defined by the occurrence of distinct oncogenic driver mutations are mostly characterized by certain phenotypic characteristics like female gender, adenocarcinoma histology and never-smoking status in *EGFR*-mutated or *ALK*-translocated NSCLC or squamous cell histology and heavy-smoking history in *FGFR1*-amplified NSCLC [7]. By comparison, no such characterization has been reported so far for *PIK3CA*-mutated NSCLC.

Here we present the genetic and phenotypic analysis of a *PIK3CA* mutated subgroup within a cohort of 1144 NSCLC patients consecutively collected over a period of two years in a molecular screening network for lung cancer.

## RESULTS

### Frequency and clinicopathologic features of *PIK3CA*-mutated patients in NSCLC

**Figure 2:**
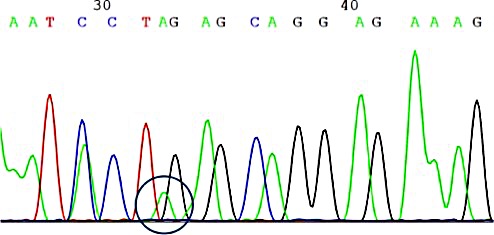
Detection of a *PIK3CA* mutation detected by dideoxysequencing The example here shows a 74 year-old male patient with detection of Exon 9 c.1633G>A point mutation, leading to p.E545K substitution. The patient had a history of renal-cell carcinoma and bladder cancer in the past. NGS revealed an additional *HRAS* p.G12D mutation.

FFPE tissue from 1144 patients was checked for *PIK3CA* mutations. Of these, 859 (75.1%) had adenocarcinoma histology (AD), 179 (15.6%) had squamous cell carcinoma histology (SQCC), and 106 (9.3%) had other histological subtypes or could not be precisely characterized due to insufficient tissue quality (n=57, 5.0%). We detected in 42 patients (3.7%) mutations within exon 9 or exon 20 of the *PIK3CA* gene (Figure 2). Of these patients, 25 patients (59.5%) had AD, and 16 (38.1%) had SQCC histology. One patient (2.4%) was classified as adenosquamous carcinoma. Thus, the frequency of *PIK3CA* mutations in SQCC was 8.9% (16/179) and in AD 2.9% (25/859), thereby significantly higher in patients with SQCC (p<0.001, odd's ratio 3.27, Fisher's exact test).

Among the *PIK3CA* mutated patients, 25 (59.5%) were male, whereas 17 (40.5%) were female. 13 patients (31.0%) had stage IV NSCLC, stage III was present in 15 cases, two patients had stage II, and 12 patients presented with stage I. One of 38 evaluable patients (2.7%) had a G1 grading, 17 (44.7%) G2 and 20 (52.6%) G3. In total, 38 of 42 samples (90%) derived from lung tumors. From the 13 stage IV patients, in 9 (69.2%) diagnostic biopsy was taken from the main lung tumor, whereas from the remaining 4 patients, in 2 diagnostic biopsy was taken from ipsilateral pleura parietalis and in the other 2 from initial brain metastases.

Of 38 evaluable patients, 35 patients (92.1%) had a smoking history, being either former (n=11, 28.9%) or current (n=24, 63.2%) smokers, whereas 3 patients (7.9%) met the criteria of being never-smokers. To compare smoking status with *PIK3CA* wildtype patients, we analyzed a group of 211 patients (comprising 71 patients with *FGFR1* amplification, 17 with *BRAF* mutation, 17 with *ALK* translocation, 46 with *EGFR* mutation, 37 with *KRAS* mutation, and 23 patients without detected genetic aberration) of which we had the same clinical annotation (33.6% SQCC, 66.4% AD) without *PIK3CA* mutation. By comparison with this group, patients with *PIK3CA* mutation had a significantly higher exposure to smoking (p=0.041, Chi square, and p=0.012, trend test). We also built subgroups of patients with aberrations known to correlate with smoking status. Patients with *PIK3CA* mutation had a significant higher exposure to smoking compared to patients with *EML4-ALK* translocation or *EGFR* mutation known to have a negative correlation with smoking (n=63, p<0.001, Chi square), but a lower exposure compared to patients with aberrations described as smoking-associated (*FGFR1* amplification and *KRAS* mutation, n=108, p=0.003, Chi square). In contrast, there was no significant difference of smoking history between patients with *PIK3CA* mutation and patients without a detected genetic aberration (n=23, p=0.151).

**Figure 3:**
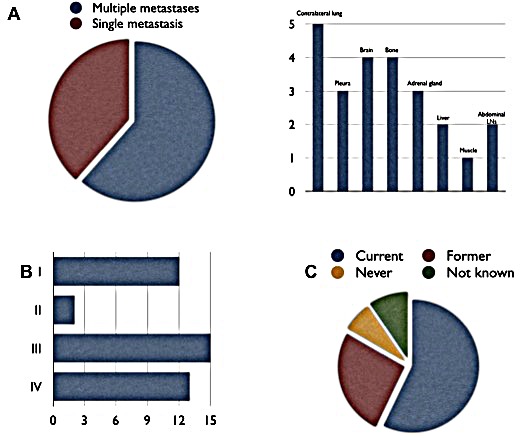
Clinical presentation of patients harboring *PIK3CA* mutations A: Metastatic pattern and tissue distribution of the metastases. B: Frequencies of different UICC stages. C: Smoking status of the patients.

Of the 13 stage IV patients, 5 (38.5%) had only one additional site of metastasis, whereas multiple sites were affected in the remaining 8 patients (61.5%). Two patients (15.4%) had more than two additional sites of metastases (see Figure 3).

A listing of the patient characteristics is shown in Table 1.

**Table 1:** Clinicopathological characteristics of patients harboring *PIK3CA* mutations (n=42)

Characteristics	Number of patients		%
Age at diagnosis, years Mean Standard Deviation Median Range	42	68.0 8.0 69.5 48-82	100
Gender Women Men	17 25		40.5 59.5
Smoking Never Former Current Not known	3 11 24 4		7.1 26.2 57.1 9.5
Histology Adeno Squamous cell Other	25 16 1		59.5 38.1 2.4
UICC tumor stage I II III IV	12 2 15 13		28.6 4.8 35.7 31.0
Grading G1 G2 G3 Not known	1 17 20 4		2.4 40.5 47.6 9.5
Stage IV patients One metastasis - pulmonal - cerebral Multiple metastases - pulmonal - pleural - cerebral - bones - adrenal - hepatic - muscle - abdominal LNs	13 5 3 2 8 2 3 2 4 3 2 1 2		38.5 61.5

### *PIK3CA* mutations and co-occurrence of other genetic aberrations

**Figure 4:**
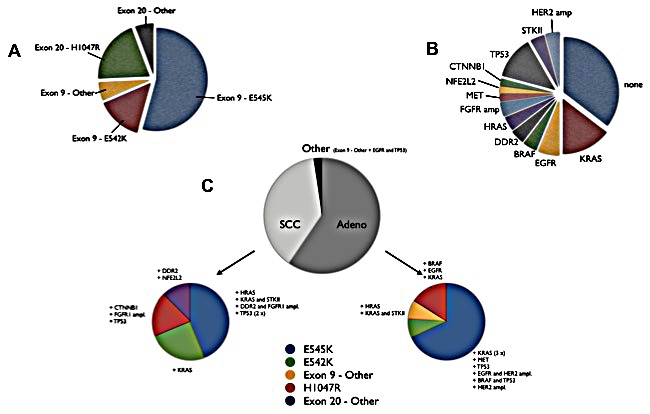
Results of mutational analyses A: Distribution of different mutations in *PIK3CA*-mutated NSCLC. B: Additional mutations and their distribution found in the patients. C: Occurrence of mutations depending on the underlying histology.

Of the 42 identified mutations, 24 (57.1%) were exon 9 E545K mutations (see Figures 2 and 4) and 6 (14.3%) exon 9 E542K. Three patients had rare mutations in exon 9 (P539L, P539R, and E545Q). Taken together, exon 9 mutations were present in 78.6% among *PIK3CA*-mutated patients. The remaining mutations affected exon 20: 7 patients (16.7%) had a H1047R mutation. One patient had H1047L, and one patient presented with a not yet described M1055L mutation (a point mutation of c.3163A>C, confirmed by NGS) within exon 20. Figure 4A shows the distribution of the *PIK3CA* mutations among the patients. A line-listing of the mutations is given in Table 2.

**Table 2:** Genetic characteristics of patients with NSCLC harboring *PIK3CA* mutations (n=42)

Characteristics	Number of patients	%
Exon Exon 9 Exon 20	33 9	78.6 21.4
Type of mutation Exon 9 – E545K Exon 9 – E542K Exon 9 – other Exon 20 – H1047R Exon 20 – other	24 6 3 7 2	57.1 14.3 7.1 16.7 4.8
Additional aberration No Yes - detected in standard panel only - detected in standard panel and *NGS*	18 24 7/20 17/22	42.9 57.1 35.0 77.3
Types of aberration *KRAS* *BRAF* EGFR* FGFR1 Ampl.** DDR2** *HRAS* *NFE2L2* *CTNNB1* *MET* TP53*** HER2neu Ampl.* STK11^****^	7 2 2 2 2 2 1 1 1 3 2 2	16.7 4.8 4.8 4.8 4.8 4.8 2.4 2.4 2.4 7.1 4.8 4.8

* One patient with *EGFR* mutation had *HER2* amplification, too.

** One patient had both *DDR2* mutation and *FGFR1* amplification.

*** One patient with *TP53* mutation had an *EGFR* mutation, respectively, and is listed there. Four single-nucleotide-polymorphisms (P72R) are not listed here.

Of 20 patients (47.6%) without NGS analysis, our standard diagnostic molecular marker panel revealed additional mutations in 7 patients (35.0%) (see Table 2 and Figure 4B). NGS was performed in 22 patients, whereof 17 patients (77.3%) harbored additional genetic aberrations. The detected additional mutations were: 7 *KRAS* (G12V, G12F, Q61L, 2x G12C, 2x G12D), 2 *BRAF* (V600E, G596R), 2 *EGFR* (L747_P753delinsS, Q791H), 2 *DDR2* (R473P, V302L), 2 *HRAS* (G12D, G12_G13delinsVF), 1 *MET* (M1229L), 1 *CTNNB1* (P44A), 1 *NFE2L2* (G31A), and 2 *STK11* (I29M, S404F). *HER2* and *FGFR1* amplification were detected twice each. In three of out of 9 analyzed samples (33.3%), we found relevant mutations within the *TP53* gene (G199V, E285Q, E298*), besides four P72R polymorphisms. The occurrence of additional genetic aberrations did not differ significantly between the histological subgroups (Chi square, p=0.40). *EGFR* mutations were not detected in SQCC, whereas *KRAS*, *HRAS* and *STK11* mutations occurred in both SQCC and AD. Figure 4C shows the distribution of *PIK3CA* mutations and the additional aberrations in the different histological subgroups.

### Prior malignancies in *PIK3CA*-mutated NSCLC patients

In our *PIK3CA*-mutated cohort, 18 patients (42.9%) suffered from NSCLC occurring as a secondary malignancy. Table 3 lists the respective patients. NSCLC occurred with a median of 8 years (range, 1 to 25 years) after initial diagnosis of the preceding cancer. 4 patients (22.2%) did neither have chemotherapy nor radiation therapy in the treatment of the initial neoplasm. Detection of mutations of the initial tumor was not covered by the ethics vote, and therefore could not be assessed. Of the 18 patients, 11 (61.1%) had additional genetic aberrations in their NSCLC tumor samples.

**Table 3:** NSCLC with *PIK3CA* mutation as a secondary malignancy. Characteristics of patients with a history of cancer in the past prior to diagnosis of NSCLC (n=18)

ID	Gender	Age	Histology, PIK3CA mutation	Additional genetic aberration	Primary malignancy (PM)	Diagnosis of PM	Treatment of PM
01	m	56	AD, E545K	-	Hodgkin lymphoma	1990	RCTX
10	f	70	AD, E545K	TP53	Breast cancer	2007	OP, adjuvant RTX
11	m	82	SCC, E542K	-	CRC	2002	neoadjuvant RCTX, OP
14	f	70	AD, H1047R	-	Endometrial Ca, Ovarial-Ca	1996	OP, adjuvant CTX
15	f	73	AD, E545K	EGFR, HER2neu ampl.	CRC	2010	OP
17	f	67	AD, H1047L	DDR2	Breast cancer	2002	OP, adjuvant RCTX
18	f	64	SCC, E545K	TP53 (SNP)	Non-Hodgkin Lymphoma	1989	multiple CTXs
21	m	77	AD, E545K	-	Urothel-Ca, CRC	2003, 2009	OP (both)
23	f	69	SCC, H1047R	FGFR1 ampl.	Breast cancer	2007	OP, adjuvant RTX
24	m	67	AD, E545K	-	NSCLC (SCC)	1994	OP
25	m	74	SCC, E545K	HRAS	RCC, Bladder-Ca	2003, 2006	OP (RCC), TUR + local Mitomycin (Bladder)
26	f	62	AD, H1047R	-	Breast cancer	1986	OP (1986), RCTX (1990), Tamoxifen (2001-2006)
27	f	59	AD, H1047R	KRAS	NSCLC (SCC)	2007	OP, RCTX
31	f	70	SCC, E545K	KRAS, STK11	Cervix-Ca	1997	OP, adjuvant RTX
34	m	72	SCC, E542K	KRAS	NSCLC (AD)	2008	OP, adjuvant RCTX
35	m	64	SCC, E545K	FGFR1 ampl.	HNSCC	2010	OP, adjuvant RTX
39	f	63	SCC, E542K	-	Breast cancer	2004	OP, RCTX, Tamoxifen
42	m	78	AD, E545Q	KRAS, STK11	RCC	2002	OP

m: male, f: female; ampl.: gene amplification; RCTX: Combined radiation with chemotherapy, RTX: Radiation, CTX: Chemotherapy, OP: Surgery. TUR: transurethral resection. CRC: Colorectal carcinoma, RCC: Renal cell carcinoma, HNSCC: Head-and-neck squamous cell carcinoma, Ca: Cancer. AD: Adenocarcinoma, SCC: Squamous cell carcinoma.

Of the primary tumors, 5 (27.8%) were breast cancers, whereof 3 had exon 20 mutation in the NSCLC. In three cases, the patients had lung cancer of a different histology in the past. 3 patients (16.7%) had two different tumor types in their history. We compared these findings with the 211 *PIK3CA*-wildtype patients mentioned above. 35 of these patients (16.6%) had a history of cancer in the past. Thus, *PIK3CA*-mutated patients had significantly more often a cancer history (p<0.001, Chi square).

As expected, patients with NSCLC as a secondary tumor were older when diagnosed than patients without cancer history (mean 70.1 years [SD, 8.1] vs 65.0 years [SD, 10.4], p=0.001). As *PIK3CA-*mutated patients were not significantly older than the comparing group (mean 68.0 years [SD, 8.0] vs 65.7 years [SD, 10.5 years], p=0.176), we performed a multivariable logistic regression analysis with age and *PIK3CA* mutation regarding the occurrence of additional cancer within the past. Here, *PIK3CA* mutation and age proved to be independent predictive factors (p=0.001 and p=0.002, respectively) for the occurrence of NSCLC as a second malignoma. Nevertheless, age and the presence of a mutation were not correlated to each other (p=0.176, Pearson's correlation).

### Clinical outcome and survival

**Figure 5:**
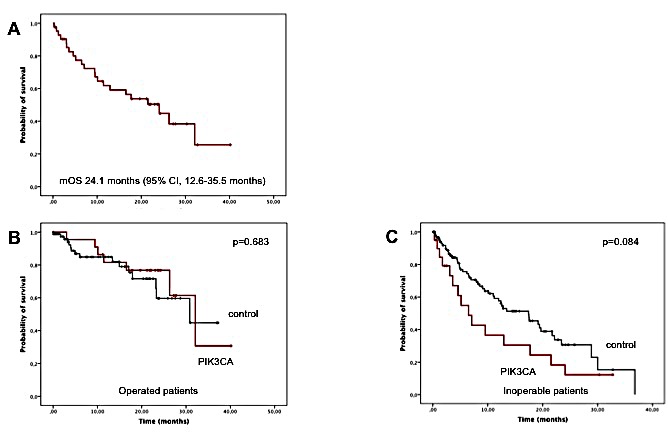
Results of survival analyses for *PIK3CA*-mutated patients A: All patients (n=42). B: Operated patients with *PIK3CA* mutation compared with the operated control group (n=86). C: Inoperable patients with *PIK3CA* mutation compared with the inoperable control group (n=125).

The median follow-up for patients being still alive when the database was closed was 22.1 months. Median OS was 24.1 months (95% CI, 12.6 – 35.5 months), with 20 patients (47.6%) still alive (see Figure 5A).

22 patients (52.4%) received local therapy with curative intention. As expected, their survival was significantly longer compared to patients who received palliative systemic treatment approaches (median OS, 32.1 months [95% CI, 23.6-40.6 months] vs 6.5 [3.3 - 9.7 months], p=0.001, log rank). Of the 22 patients, 9 received no further treatment after R0 resection, 5 had adjuvant chemotherapy, 3 received adjuvant radiation therapy, and 5 received both chemotherapy and radiation. Of the non-resected tumor patients (n=20), 4 did not receive any systemic treatment approach (3 stage IIIB, 1 stage IV) due to local complications. 2 patients received symptomatic radiation of single metastases, and two patients had local radiation therapy after platin-based chemotherapy. The remaining 12 patients had systemic treatment with at least two cycles of chemotherapy or continuous oral anticancer medication.

Best response in the stage IV group was a complete response (ongoing for 2.5 years) in a female patient harboring a H1047R mutation without an additional genetic aberration and receiving erlotinib and bevacizumab. Also a second patient with E545K and being treated with erlotinib and bevacizumab had no further genetic aberration and stable disease for 12.9 months. A female patient with an additional *KRAS* mutation and relapse after resection of her stage I tumor had repeatedly responded to therapy with pemetrexed. There was no reported response to platinum-based therapy. Median OS in patients who received systemic treatment was 9.5 months (95% CI, 2.3 – 16.8 months) and 32.1 months (95% CI, 17.9 – 46.3 months) for the local stages.

The operated patients did not differ significantly from the operated control group (n=86, 30.9 months [95% CI, 16.1- 45.6 months], p=0.683) (Figure 5B). In comparison with patients without detected mutations and surgery (n=9), *PIK3CA*-mutated patients did not differ significantly regarding OS, as all data was censored for the mutation-negative group (p=0.582). As there were also not enough events in the operated *KRAS*-mutated, BRAF-mutated, and EGFR-mutated patients, no statistics regarding these subgroups of the control group could be performed. In comparison with PIK3CA-mutated patients, non of the subgroups had a significantly different mOS (see Table 4A).

**Table 4:** Comparison of *PIK3CA*-mutated patients with the respective control group depending on genetic aberrations. A: operable patients, B: inoperable patients

A			
Aberration	n	mOS (95% CI)	log rank vs PIK3CA
*FGFR1*_amp_	39	30.9 (20.0-41.7)	0.480
*BRAF*_mut_	5	n/a	0.428
*ALK*_translocation_	6	23.2 (13.4-33.0)	0.321
*EGFR*_mut_	22	n/a	0.684
*KRAS*_mut_	4	n/a	0.656
no mutation	9	n/a	0.582
all	86	30.9 (16.1-45.6)	0.683

B			
Aberration	n	mOS (95% CI)	log rank vs PIK3CA
*FGFR1*_amp_	32	11.0 (5.6-16.4)	0.913
*BRAF*_mut_	12	10.1 (4.1-16-0)	0.338
*ALK*_translocation_	11	12.5 (7.7-17.3)	0.560
*EGFR*_mut_	24	28.9 (22.0-35.7)	<0.001^*^
*KRAS*^mut^	32	6.6 (1.2-12.0)	0.812
no mutation	14	17.4 (0-30.0)	0.175
all	125	17.4 (11.4-23.5)	0.084

For inoperable patients, OS did not significantly differ for the *PIK3CA*-subgroup as compared with mutation-negative patients (n=14, 6.5 [95% CI, 3.3 - 9.7] months vs 17.4 [95% CI, 0 - 38.0] months. P=0.175, log rank). Compared with the whole control group not operated, *PIK3CA*-mutated patients had a tendency to worse outcome, even though not significant (n=125, 17.4 [95% CI, 11.4 - 23.5 months], p=0.084, log rank) (Figure 5C). This tendency was due to the high number of *EGFR*-mutated patients, as these patients were the only subgroup with a significantly better OS for non-operated patients regarding *PIK3CA*-patients (28.9 [22.0 - 35.7] months, p<0.001, log rank). Compared with the other subgroups, there was no difference in survival for patients with *PIK3CA* mutation (Table 4B).

## DISCUSSION

Here we provide a detailed characterization of *PIK3CA*-mutated NSCLC, to our knowledge in the largest cohort of this genetically defined lung cancer subgroup reported so far.

In our cohort, the prevalence of *PIK3CA* mutations was 3.7% confirming the published prevalence of about 2-4% in NSCLC [7, 25]. Although mutations were found in AD and SQCC, the prevalence in SQCC was signifantly higher with 8.9% compared to 2.9% in AD, thus exceeding the frequency reported earlier for SQCC in smaller cohorts substantially [24]. The frequency of adenocarcinoma in our cohort (75.1%) was roughly representative for Caucasian NSCLC cohorts, however sqamous cell carcinoma was slightly underrepresented (15.6%). While this does not influence our results for the histology-dependent frequencies of PIK3CA mutations, their frequency in the total population of NSCLC patients might be slightly higher than 3.7%. Comparison with a *PIK3CA* wildtype cohort revealed a strong association with smoking. However, no further specific phenotypic characteristics concerning gender distribution, stage, metastatic spread or overall survival were found. The overall survival (OS) analyses showed no negative impact of *PIK3CA* mutations with the exception of *EGFR*-mutated patients treated systemically. Noteworthy, the data cut-off was in 2012, and patients with *EML4-ALK* translocation treated systemically nowadays might have a better prognosis regarding OS than the 12.5 months observed in our group.

The vast majority of *PIK3CA* mutations (78.6%) affected exon 9 as described earlier [25]. We also found one exon 20 mutation not yet described. Thus, screening of larger cohorts might reveal a more pronounced genetic heterogeneity in the future.

Co-occurrence of *PIK3CA* mutations with several driver mutations has been published recently for AD [20]. We confirm this observation and in addition show that also in SQCC *PIK3CA* mutations co-occur frequently with driver mutations namely affecting *DDR2*, *KRAS*, *EGFR*, *BRAF*, *HRAS*, *NFE2L2*, *CTNNB1*, *MET*, and *STK11*, beside amplifications of *FGFR1* and *HER2* and gatekeeper mutations within *TP53*. Thus, in analogy to the missing specific phenotypic characteristics as described above, these genotype analyses do not suggest a distinct genetic profile of *PIK3CA* mutated lung cancer neither in the AD nor in the SQCC subgroup.

With regard to optimization of molecular lung cancer diagnostics it is noteworthy that the frequency of co-occurring driver mutations was significantly higher using NGS technology (77.3%) compared to single-gene assay diagnostics (35.0%), as with NGS, a higher number of targets were screened simultaneously. This observation underlines the need of comprehensive molecular testing in order to better understand the molecular aberrations in lung cancer, as proposed recently [6, 20, 25].

An unexpected observation was that *PIK3CA*-mutated NSCLC occurs significantly more often in patients with different prior malignancies compared to *PIK3CA*-wildtype NSCLC. In our cohort there is no specific association with distinct primary tumors, as these included various malignancies of different origin including frequent adenocarcinomas such as breast and colorectal cancer but also malignant lymphomas. Further understanding of this observation, which might represent an association with long-term carcinogenic cancer treatment effects as well as with an underlying genetic predisposition requires the analysis of germline and primary tissue samples in larger cohorts. In fact, a major limitation of our work is, that we could not analyze the primary tumors genomically.

*PIK3CA* mutations have been described as one possible mechanism of resistance to EGFR-TKI therapy in *EGFR*-mutated NSCLC. In our cohort, the best response, a complete response ongoing already for 2.5 years, is seen in a patient without activating *EGFR* mutation treated first line with erlotinib and bevacizumab. Another patient without activating *EGFR* mutation showed a PFS under the same treatment regime of 12.9 months. Thus it might be worthy to analyze the role of *PIK3CA* mutations in EGFR-TKI therapy in more detail.

It has been discussed controversially, whether *PIK3CA* mutations should be regarded as driver mutations thus representing potential targets for a specific blockade of this signal transduction pathway. Up to now, PI3K-inhibitors have not yet proven to be clinically effective, at least in lung cancer, in the majority of patients with P*IK3CA*-mutation [7, 22, 26]. Our data show that *PIK3CA*-mutated NSCLC is a clincally and genetically heterogeneous group. This holds true for the AD and SQCC subgroup and strongly suggests that *PIKCA* mutations do not define a distinct lung cancer subgroup amendable to specific therapy. Rather, these mutations seem to represent passenger mutations widely distributed among the other genetically defined subgroups. The observation of the high frequency of the occurrence of *PIK3CA*-mutated lung cancer as secondary malignancy needs further investigation.

## METHODS

### Patients

Patients were diagnosed within the Network Genomic Medicine (NGM) Lung Cancer, a collaborative health care provider network for comprehensive molecular diagnostics of lung cancer encompassing 19 hospitals and 4 outpatient practices in the wider catchment area of the University Hospital of Cologne, Germany. Within NGM all participating centers send formalin-fixed paraffin-embedded (FFPE) lung cancer samples to a central laboratory for genotyping. We analyzed incoming samples in a predefined time-frame of two years from March, 2010 until March, 2012.

### Specimen collection and molecular diagnostics

The study has been reviewed by the Ethics Committee of the University of Cologne. Diagnostics were performed centrally according to local standard operating procedures. The histopathological differentiation between AD and SQCC was performed according to recently defined parameters[27], using CK7, CK5/6, TTF1, PAS, and p63 staining. As part of the routine genotyping procedure in our network (standard panel), patients with AD were screened for *KRAS* (exons 2 and 3), *BRAF* (exon 15) and *PIK3CA* (exons 9 and 20) mutations using high resolution melting (HRM) curve analyses and positive samples were then confirmed by dideoxy (“Sanger”) sequencing. Next, direct Sanger sequencing of the *EGFR* gene (exons 18, 19, and 21) and break-apart FISH for *EML4-ALK* was performed. In patients with SQCC, we screened for *KRAS*, *BRAF* and *PIK3CA* in the corresponding exons and for *FGFR1* amplifications with FISH [28]. In patients with *PIK3CA* mutations and enough tumor material left for further evaluation, next-generation sequencing (NGS) was performed using Illumina MiSeq system (Illumina, San Diego, California). More details about the panels used in NGS are given as supplement. In a subset of *PIK3CA*-mutated patients, *HER2*-amplification and mutation status was also checked.

### Staging

All patients underwent local standardized staging procedures using CT scans, MRI scans of the brain, and bone scintigraphy, if indicated. In a subset of stage IV patients (n=4), FDG-PET-CT scans were performed.

### Clinical parameters

Age, gender, grading and tumor stage at diagnosis were assessed. For grading and staging, we used the UICC classification. Smoking status, medical history including concomitant diseases and cancer history was reported. Stage IV patients with *PIK3CA*-mutation were further analyzed regarding their metastatic sites. Smoking status was set as follows: Patients with less than 100 cigarettes in their lifetime were considered never smokers, patients with more than 100 cigarettes who quit smoking at least one year before first diagnosis of lung cancer were considered former smokers, and patients with a smoking history of more than one pack-year who continued smoking for a period shorter than one year before diagnosis were considered current smokers. In order to warrant comparability, cancer history and smoking status were assessed in a subset of *FGFR1*-amplified, *EGFR*-mutated, *KRAS*-mutated, *BRAF*-mutated, *EML4-ALK* translocated tumors and tumors negative for all these markers. In *PIK3CA*-mutated patients, survival was determined from the date of first diagnosis. Data cut-off regarding survival was July 22^th^, 2013.

### Statistical analysis

Qualitative variables were summarized by count and percentage, quantitative variables (i.e. age) by mean, standard deviation, median and range. Distribution of time to event was described by the Kaplan-Meier curve and compared between groups by the log-rank test. Association of qualitative variables were tested for by chi-square or Fisher's exact test, contingent on distributional assumptions. Overall survival (OS) was defined as the time period from the date of first diagnosis until death. Patients who were still alive at the data cut-off were censored. For multivariable analysis, a multiple logistic regression was performed. Bivariable association with smoking was evaluated by the chi-square test for trend.
